# Tanning Wastewater Treatment by Ultrafiltration: Process Efficiency and Fouling Behavior

**DOI:** 10.3390/membranes11070461

**Published:** 2021-06-22

**Authors:** Fu Yang, Zhengkun Huang, Jun Huang, Chongde Wu, Rongqing Zhou, Yao Jin

**Affiliations:** 1 College of Biomass Science and Engineering, Sichuan University, Chengdu 610065, China; yangfu19960513@163.com (F.Y.); huangzhengkun969@163.com (Z.H.); Huangjun@scu.edu.cn (J.H.); wuchongde@163.com (C.W.); zhourqing@scu.edu.cn (R.Z.); 2 Key Laboratory for Leather and Engineering of the Education Ministry, Sichuan University, Chengdu 610065, China; 3 National Engineering Research Center of Solid-State Manufacturing, Luzhou 646000, China

**Keywords:** tanning wastewater, ultrafiltration, membrane fouling, fouling propensity, pore blocking

## Abstract

Ultrafiltration is a promising, environment-friendly alternative to the current physicochemical-based tannery wastewater treatment. In this work, ultrafiltration was employed to treat the tanning wastewater as an upstream process of the Zero Liquid Discharge (ZLD) system in the leather industry. The filtration efficiency and fouling behaviors were analyzed to assess the impact of membrane material and operating conditions (shear rate on the membrane surface and transmembrane pressure). The models of resistance-in-series, fouling propensity, and pore blocking were used to provide a comprehensive analysis of such a process. The results show that the process efficiency is strongly dependent on the operating conditions, while the membranes of either PES or PVDF showed similar filtration performance and fouling behavior. Reversible resistance was the main obstacle for such process. Cake formation was the main pore blocking mechanism during such process, which was independent on the operating conditions and membrane materials. The increase in shear rate significantly increased the steady-state permeation flux, thus, the filtration efficiency was improved, which resulted from both the reduction in reversible resistance and the slow-down of fouling layer accumulate rate. This is the first time that the fouling behaviors of tanning wastewater ultrafiltration were comprehensively evaluated, thus providing crucial guidance for further scientific investigation and industrial application.

## 1. Introduction

The wastewater produced by the leather industry is considered to be one of the most contaminated wastes, since there is a considerable amount of organic material (mainly dissolved fat, protein, keratin, etc.) and inorganic chemicals (various salts, such as chloride Sodium, sodium sulfate, calcium hydroxide, sodium sulfide, etc.). The presence of these substances leads to high chemical oxygen demand (COD), biochemical oxygen demand (BOD), suspended solids (SS), conductivity, and so on. In this process, a large amount of organic and inorganic chemicals are discharged, causing widespread water and soil pollution [1]

There are many ways to minimize the pollution of tannery wastewater, such as electrocoagulation [2], chemical coagulation [3], advanced oxidation processes [4], adsorption process [5], membrane processes [6], biological treatment [7], and ion exchange [8]. Compared with other concentration and separation methods, the main advantage of the membrane treatment is that concentration and separation can be achieved, in most instances, without the use of chemicals or thermal energy and a state change [9], and membrane technology is ecologically friendly.

In fact, there are already several investigations on the use of membrane technology to treat tanning wastewater in the literature. The reuse of permeate was reported in a study implemented in a pilot plant using ultrafiltration tubular inorganic membranes [1]. Other authors have reported good results when using nanofiltration (NF) to treat these wastewaters, despite the operating pressure, is extreme [10,11]. There are also reports on the use of a membrane bioreactor (MBR) to treat tannin effluents [12]. Up to now, there are reports that membrane technology has been successfully applied to the treatment of wastewater generated in different stages of the leather industry. For instance, ultrafiltration (UF) was applied to the unhairing, tanning, and dyeing stage [13,14,15], and nanofiltration and Reverse Osmosis were applied to the chrome tanning stage [16]. Hence, membrane separation has shown to be a promising water reuse technology to achieve zero liquid discharge (ZLD) in the leather industry.

However, the current studies mainly focus on the removal efficiency of contaminants and the analysis of membrane flux. Few revealed the filtration behavior of such a process and the corresponding fouling mechanism. Better understanding of the filtration behavior is crucial for process optimization and intensification, which is necessary for its large-scale application in wastewater treatment in the leather industry.

Membrane fouling is mainly caused by the deposition of particles inside the membrane pore and/or the formation of a cake/gel layer on the membrane surface, which leads to an increase in filtration resistance, which, in turn, results in attenuation of permeation flux and ultimately reduces the life of the membrane [17,18]. The formation of fouling depends on various parameters, such as operating parameters [19,20], membrane material [21,22], and feed characteristics [23,24]. Therefore, it is necessary to take into account the abovementioned parameters to investigate the fouling behavior.

From those considerations, this work employed UF, as an upstream process of a ZLD system, to treat the mixed effluents from the tannin stage of leather production. The permeation efficiency, removal efficiency, filtration resistance, fouling propensity, and pore blocking mechanisms of the ultrafiltration process were evaluated under different operating conditions (shear rate, transmembrane pressure) with two different membrane materials (PES and PVDF). This study provides a comprehensive understanding of membrane fouling in the ultrafiltration process of tanning wastewater and provides guidance for its industrial application.

## 2. Materials and Methods

### 2.1. Characteristics of Tanning Wastewater

The tanning wastewater was acquired from a leather-producing process, which was gathered in National Engineering Laboratory for Clean Technology of Leather Manufacture (Sichuan University), located in the Sichuan Province of China. The effluent was stored at 4 °C and returned to room temperature before use. Table 1 summarizes the physical-chemical parameters of the tanning wastewater, each sample was measured in triplicate to ensure good repeatability, and the data was presented in the form of mean ± relative standard deviation (RSD).

The characteristics of the tanning wastewater were carried out by the measurement of pH and electrical conductivity using a multiparameter analyzer (DZS-708-A, Leici, Shanghai, China). Chemical oxygen demand (COD) was determined with a detector (COD-571, Leici, Shanghai, China). Fat was determined by dichloromethane extraction. Suspended solids (SS) were measured by the Filter Method, Cellulose acetate membrane filters (0.45 μm, 50 mm of diameter) were used [25]. The determination of the concentration of fat and suspended solids are calculated gravimetrically. Protein was determined by means of an ultraviolet-visible spectrophotometer (L7, Leici, Shanghai, China).

### 2.2. Ultrafiltration Membranes and Experimental Setup

Two distinct types of ultrafiltration commercial membranes were tested and compared: PES 50 kDa and PVDF 50 kDa. Which were used in a lab-scale ultrafiltration stirred cell (MSC300, Mosu, Shanghai, China) to filter the effluent. The details of the two membranes, according to the manufacturer are shown in Table 2.

**Figure 1:**
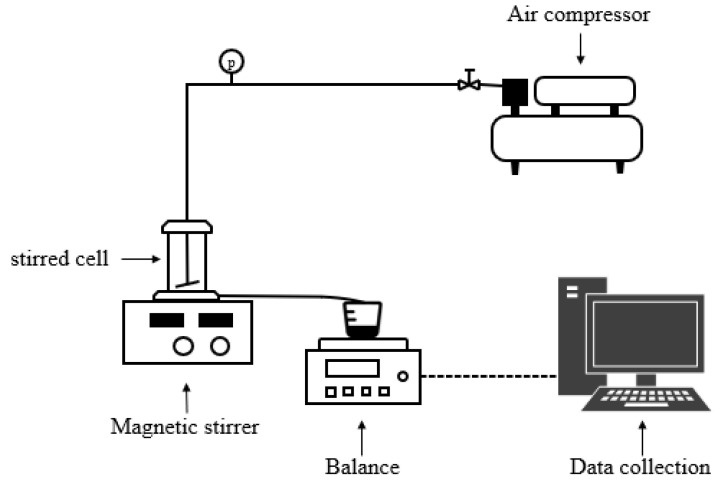
Layout of ultrafiltration setup.

Membrane experiments were conducted in a laboratory-scale device, the diagrammatic drawing of the ultrafiltration set up as shown in Figure 1. Experimental setup was equipped with a magnetic stirrer to achieve the operating shear rate. The operating pressure was applied by air compressor. The temperature of experimental hall was maintained constantly at 20 °C by air-conditioner. The permeate stream was collected by a digital balance (CP4102, Ohaus, Co., Ltd., Parsippany, NJ, USA) and recorded every 10 s with an accuracy of 0.01 g via the data collection application (SPDC, Version 2.01, Parsippany, NJ, USA). Each experiment was repeated at least three times to ensure accuracy.

Assays were performed at four different shear rates (5.6 × 10^2^ s^−1^, 2.9 × 10^3^ s^−1^, 6.3 × 10^3^ s^−1^, 9.3 × 10^3^ s^−1^) and transmembrane pressures (0.6 bar, 0.8 bar, 1.0 bar, 1.2 bar). The UF cup used was cylindrical, made of polymethyl methacrylate, with an effective filtration area of 0.0015 m^2^. The volume of the UF cup is 300 mL, prior to employ, the polymer films were continuously immersed in ultrapure water for 24 h to remove impurities or additives in the production process, and the membranes were washed and compacted with ultrapure water prior to measurements. The compaction step allows a stable membrane structure to be obtained. In the meantime, the filtrate guide pipe is filled by ultrapure water, which avoids the data not being recorded in the initial filtration. Each membrane was used for a set of tests under given operating conditions (for example, the set of TMP or shear rates). After each experiment with the tanning wastewater, the fouled membrane was cleaned with 0.5 mol/L sodium hydroxide solution and citric acid solution for 30 min. The cleaned membrane is kept moist in 0.5% sodium hydrogen sulfite solution to prevent bacteria from growing on the membrane.

### 2.3. Calculated Parameters

The shear rate, also called velocity gradient on the membrane surface, was calculated according to Tang et al. [26]

The hydraulic permeability coefficient ($L_{P}$) of the membrane was Calculated by measuring the water flux ($J_{w}$) under different TMP (Equation (1)).

(1) $$L_{P}=\frac{J_{w}}{∆P}$$

The separation performance of the UF membranes by evaluating the removal efficiency (R, %) for COD, fat, SS, and protein, according to:

(2) $$R=\left(1-\frac{c_{p}}{c_{f}}\right)\times 100\%$$

where $c_{p}$ and $c_{f}$ represented the concentrations of the water quality physicochemical indexes in permeate and feed, respectively. In order to ensure accuracy, at least three parallel experiments were performed.

### 2.4. Models of Membrane Fouling Analysis

#### 2.4.1. Resistance-In-Series Model

The permeate flux can be calculated in the light of Darcy’s law (Equation (3)):

(3) $$J=\frac{∆P}{\mu R_{t}}$$

where  J is the permeate flux (m^3^·m^−2^·s^−1^), ∆P is the transmembrane pressure (Pa), μ is the dynamic viscosity of the feed liquid (Pa·s) and $R_{t}$ is the total resistance (m^−1^), which is shown in Equation (4).

(4) $$R_{t}=R_{m}+R_{f}=R_{m}+R_{rf}+R_{irrf}$$

$R_{m}$ represents the intrinsic membrane resistance, the fouling layer resistance ($R_{f}$) is calculated as the sum of the reversible resistance ($R_{rf}$), caused by concentration polarization and cake fouling onto the membrane surface and the irreversible resistance ($R_{irrf}$), caused by attachment of compounds on membrane surface or into the pores. $R_{rf}$ can be removed by simple cleaning, $R_{irrf}$ is hardly removed by physical cleaning inversely [27]. Equations (5)–(7) were used to calculate each item.

(5) $$R_{m}=\frac{∆P}{\mu_{0}J_{0}}$$

(6) $$R_{f}=R_{t}-R_{m}=\frac{∆P}{\mu J}-\frac{∆P}{\mu_{0}J_{0}}$$

(7) $$R_{rf}=R_{f}-R_{irrf}=\frac{∆P}{\mu J}-\frac{∆P}{\mu_{0}J_{1}}$$

where $J_{0}$ is the permeate flux filtrating ultrapure water, $J_{1}$ is the water flux after removing the superficial cake layer with ultrapure water, $\mu_{0}$ is the dynamic viscosity of ultrapure water.

#### 2.4.2. Membrane Fouling Propensity Model

Using the model to evaluate the growth of fouling, the total resistance of the filter was described by Equation (8) [28].

(8) $$R_{t}=R_{m}e^{K_{0}V/A}\;$$

where $K_{0}$, V, and A represent the exponential fouling coefficient (m^−1^), the filtration volume (m^3^) and the filtration area (m^2^) respectively. $K_{0}$ is an empirical constant, which indicates the growth rate of filtration resistance.

Equation (9) is the general filtration mathematical expression [29]:

(9) $$J=\frac{1}{A}\frac{dV}{dt}=\frac{∆P}{\mu R_{t}}$$

where *t* stands for filtering time (s).

Replacing $R_{t}$ of Equation (9) with Equation (8) to acquire Equation (10):

(10) $$\frac{1}{A}\frac{dV}{dt}=\frac{∆P}{\mu R_{m}e^{K_{0}V/A}}$$

The rearrangement of Equation (10) makes it possible to obtain Equation (11), and calculated to acquire the mathematical expression Equation (12):

(11) $$\int_{0}^{t}dt=\frac{\mu R_{m}}{∆PA}\int_{0}^{V}e^{K_{0}V/A}dV$$

(12) $$V=\frac{A}{K_{0}}\ln\left(\frac{K_{0}∆P}{\mu R_{m}}t+1\right)$$

This model has been validated in the previous analysis of membrane fouling [30,31,32], The value of $K_{0}$ is determined by feed category, membrane modules, operation conditions, and membrane performances. By fitting the experimental data to Equation (12), the fouling factor $K_{0}$ was calculated under different conditions in this study.

#### 2.4.3. Membrane Pore Blocking Model

The fouling type of ultrafiltration process was deduced by the Hermia blocking model [33], By the slope of the line via a linear regression fitting to the data points, the blocking mode can be easily identified (Equation (13)).

(13) $$\frac{d^{2}t}{dv^{2}}=K{\left(\frac{dt}{dv}\right)}^{n}$$

where K is a constant, and n is the blocking coefficient, the value of n determined directly using Equation (13) may be interfered by the error of the measured experimental data, that is, the experimental data of the accumulated permeation volume *v* per unit membrane area versus the time *t*. Hence, 4 ratiocinative models are usually used for linear fitting (Table 3) [33,34].

In Table 3, J is the infiltration flux (L·m^−2^·h^−1^), $J_{ini}$ is the initial infiltration flux (L·m^−2^·h^−1^), t is the filtration time (min), and $K_{b}$, $k_{s}$,$\;K_{i}$, and $K_{c}$ are the constants. In the four models, the complete blocking model assumes that the particle size is much larger than the pore size, every particle that reaches the membrane participates in the blocking, and the particles would not overlap. This situation is inconsistent with the actual situation of general filtration. The standard blocking model assumes that the membrane pores are identical cylinders, and every particle reaching the membrane surface settles onto the inner pore wall, thus resulting in a rapid drop in pore volume, which is suitable for early filtration. The intermediate blocking model assumes that every particle reaching the membrane is deposited on the surface of the particles that have reached the membrane surface or the membrane pores to participate in the blockage, which is suitable for the mid-filtration. The cake blocking model assumes that the surface and inside of the membrane are filled with particles. At this time, when the particles reach the membrane surface, they actually accumulate on the particles that have blocked pores. It is generally suitable for the filtration process after the formation of the filter cake layer. Each model is only applicable to a specific stage of membrane fouling and cannot fully express the entire process.

## 3. Results and Discussion

Transmembrane pressure, shear rate, and membrane retention molecular weight have significant influences on the permeate flux and component removal efficiency, which are important parameters for evaluating membrane performance. Therefore, in this study, two UF membranes of different materials (PES and PVDF) were used for the UF test, and the MWCO values were both 50 kDa. Four different shear rates (5.6 × 10^2^ s^−1^, 2.9 × 10^3^ s^−1^, 6.3 × 10^3^ s^−1^, 9.3 × 10^3^ s^−1^) and transmembrane pressures (0.6 bar, 0.8 bar, 1.0 bar, 1.2 bar) were evaluated.

### 3.1. Pollutant Removal Efficiency

The wastewater properties were measured before and after each filtration experiment, and the result is expressed in terms of removal efficiency (R, %).

**Figure 2:**
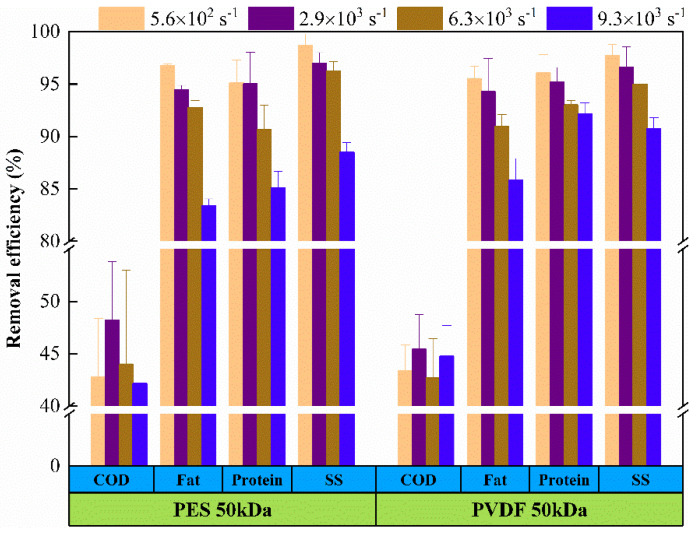
Pollutant removal efficiency of tanning wastewater ultrafiltration at different shear rates (TMP = 1.0 bar).

**Figure 3:**
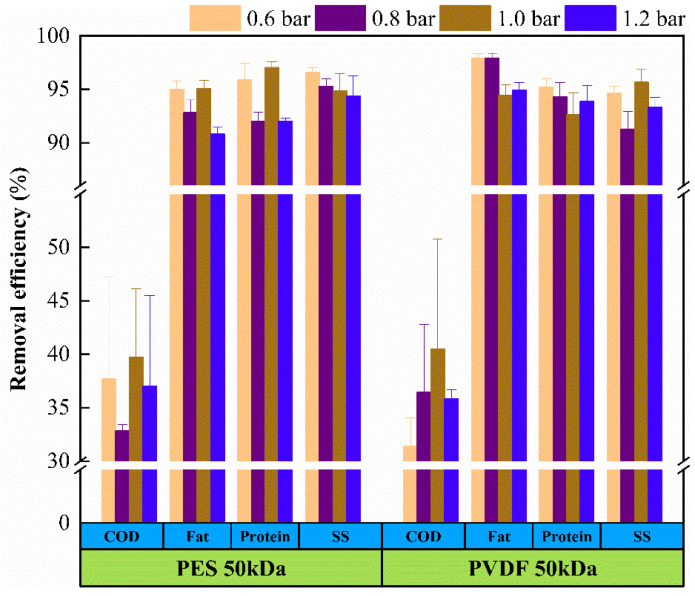
Pollutant removal efficiency of tanning wastewater ultrafiltration at different TMP (shear rate = 2.9 × 10^3^ s^−1^).

The removal efficiency observed for COD, fat, SS, and protein were calculated for the distinct UF membranes in different operating conditions. As shown in Figure 2 and Figure 3, both membranes show high removal efficiency of all the assessed components, apart from COD, the removal efficiency of SS, fat, and protein can reach more than 80%.

Figure 2 and Figure 3 demonstrate that the removal efficiency of pollutants decreased as the shear rate increased, while the change of transmembrane pressure (TMP) had little effect on the removal efficiency of pollutants. No matter what kind of membrane material, R values appeared to be the minimum at the highest shear rate, which shows that the membrane had the worst removal efficiency for pollutants under this operating condition. It can be attributed to the fact that the formation and growth of concentration polarization layer was hindered at high shear rate, the mass transfer barrier layer was thinner, and the removal efficiency of pollutants depended only on the membrane itself, so the ability of particle retention was reduced. While the change of TMP had negligible influence on the interception effect of pollutants, which shows that the influence of TMP, in the range of 0.6 to 1.2 bar, on the mass transfer barrier layer on the membrane surface was also negligible.

**Figure 4:**
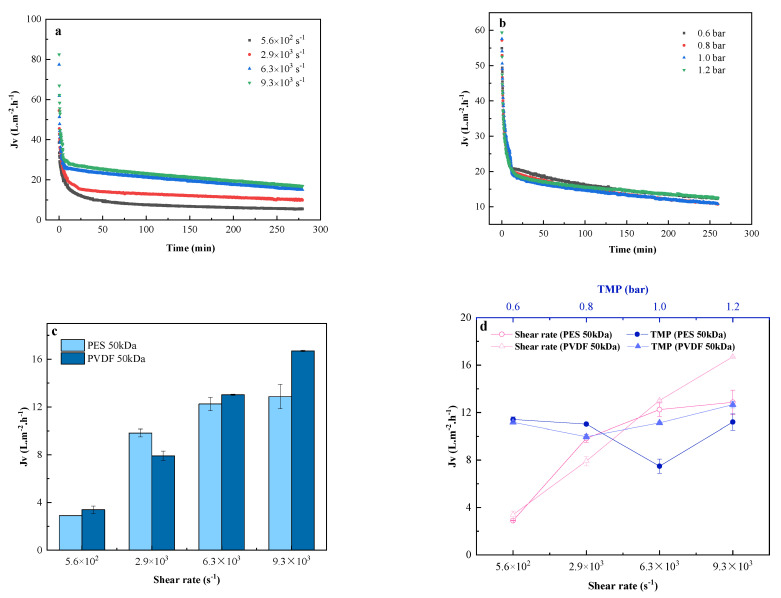
Effects of operating conditions on permeate flux of tanning wastewater ultrafiltration: (**a**) permeate flux evolution over time during UF at different shear rates (PVDF, TMP = 1.0 bar); (**b**) permeate flux evolution over time during UF at different TMP (PVDF, shear rate = 2.9 × 10^3^ s^−1^); (**c**) the steady-state permeate flux for different membrane materials; (**d**) summary of steady-state flux for various operating conditions.

The level of pollutants removal efficiency was corresponding to the measurement result of the membrane flux showed in Figure 4. That is, the removal efficiency of pollutants increased as the membrane flux decreased. The main reason for the decrease in flux was the fouling phenomenon, which is caused by the adsorption of pollutants, cake formation, and pore blockage. The trapped particles accumulated on the membrane surface to form a cake layer to reject contaminants and reduce permeation flux.

### 3.2. Permeation Efficiency

In this section, the permeation efficiency was analyzed in terms of permeate flux and cumulative infiltrate volume, revealing the influence of shear rate and transmembrane pressure on the filtration process. Previous to ultrafiltration experiments with the tanning wastewater, the membranes were compacted with ultrapure water and the permeability of the original or cleaned membranes was measured. The value of $L_{P}$ was 233.11 L/(m^2^ h bar) for PES 50 kDa and 376.72 L/(m^2^ h bar) for PVDF 50kDa. The hydraulic permeability values reported in this study are consistent with the values of similar UF polymer membranes reported in many literatures [35,36].

#### 3.2.1. Permeate Flux Evolution

Figure 4 shows the effect of operating conditions (shear rate, TMP, membrane material) on the permeate flux during tanning wastewater ultrafiltration. All filtration assays were carried out until the permeate flux reached a pseudo-steady state. Experiments show that, no matter how the membrane materials and operating conditions change, the flux dropped sharply in the initial stage and continued to decline over time. Subsequently, the flux reached a pseudo-steady state in the final stage. The operating conditions had obvious effects on the ultrafiltration process. Increasing the shear rate (Figure 4a) and TMP (Figure 4b) can effectively increase the initial flux (PVDF 50kDa was used in this study).

Figure 4a illustrates that the average permeate flux was improved to varying degrees with the increase in shear rate, which can be attributed, mainly, to the fact that the agitator greatly reduced the concentration polarization phenomenon during filtration in the dead-end filtration device. The shear force on the membrane surface was continuously increased by increasing the shear rate, and the membrane fouling was therefore attenuated. The steady state value was the lowest at 5.6 × 10^2^ s^−1^ in 4 different shear rates, suggesting that the concentration polarization was relatively serious.

Driven by TMP, the permeate flux was proportional to the transmembrane pressure, within the maximum operating pressure range that the membrane can withstand, during UF of pure water. However, during UF of tanning wastewater, TMP had no significant effect on permeate flux, the evolution trend, nor the steady-state flux, as shown in Figure 4b. Such results suggest that TMP did not alter the properties of the membrane foulants.

Figure 4c demonstrates a comparison between the steady-state flux of PES and PVDF membrane. It shows that the steady-state flux rose with the increase in the shear rate for both membranes without significant difference between them under the same shear rate. Figure 4d illustrates a brief summary of steady-state flux of this section, confirming three statements below: 1. TMP had almost no effect on the steady-state flux; 2. No significant difference of steady-state flux between two membranes of different materials; 3. the shear rate had a strong impact on the steady-state flux, which was increased by 77.48% for PES membrane and 79.68% for PVDF membrane, when the shear rate was increased from 5.6 × 10^2^ s^−1^ to 9.3 × 10^3^ s^−1^.

#### 3.2.2. Cumulative Infiltrate Volume

The volume reduction ratio (VRR) can be used to evaluate the filtration efficiency of the membrane separation process under different operating conditions, which is calculated via $V_{0}/V_{C}$, where $V_{0}$ stands for the initial feed volume (m^3^) and $V_{C}$ stands for the retentate volume (m^3^).

**Figure 5:**
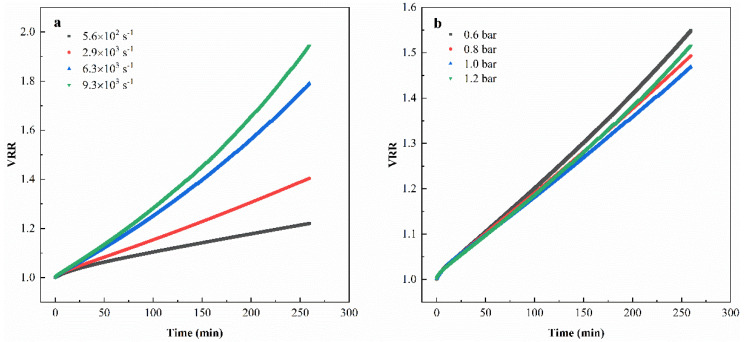
The trend of volume reduction rate (VRR) of tanning wastewater ultrafiltration with time under different operating conditions: (**a**) different shear rates (PVDF, TMP = 1.0 bar, 20 °C); (**b**) different TMPs (PVDF, shear rate = 2.9 × 10^3^·s^−1^, 20 °C).

Figure 5 shows the influence of TMP and shear rate on the trend of the volume reduction rate (VRR) of tanning wastewater ultrafiltration over time, which was carried out with the PVDF 50kDa membrane. As shown in Figure 5a, as the shear rate increased, the filtration efficiency was improved significantly. When the shear rate reached 6.3 × 10^3^ s^−1^ and above, the increase in the shear rate had a limited impact on the improvement of the filtration efficiency. Figure 5b illustrates that the filtration efficiency stayed comparable within the TMP range from 0.6 to 1.2 bar, which is consistent with the result obtained in Figure 4b.

### 3.3. Filtration Resistance

**Figure 6:**
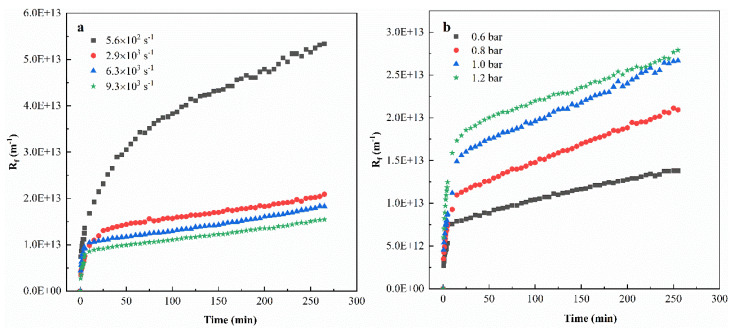

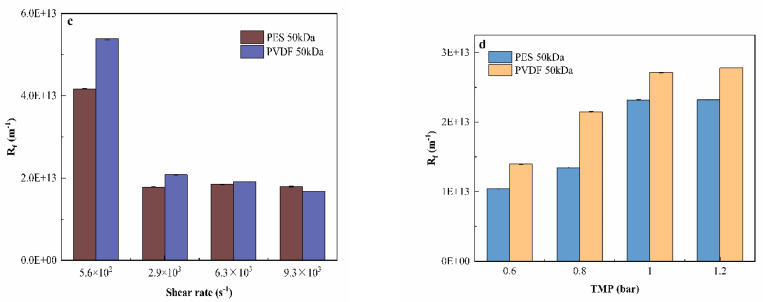
Effects of operating conditions on the process resistance ($R_{f}$) in ultrafiltration of tanning wastewater: (**a**) $R_{f}$ evolution over time during UF at different shear rates (PVDF, TMP= 1.0 bar); (**b**) $R_{f}$ evolution over time during UF at different TMP (PVDF, shear rate = 2.9 × 10^3^ s^−1^); (**c**) the steady-state $R_{f}$ for different membrane materials at different shear rates (TMP = 1.0 bar); (**d**) the steady-state $R_{f}$ for different membrane materials at different TMP (shear rate = 2.9 × 10^3^ s^−1^).

Figure 6 shows the effect of different operating conditions on the process resistance ($R_{f}$) in ultrafiltration of tanning wastewater. As shown in Figure 6a, the increase in shear rate can effectively restrain the increase in the total resistance, but the excessively high shear rate cannot reduce the total resistance of the process indefinitely. This phenomenon is similar to the change trend of membrane flux (Figure 4) and filtration efficiency (Figure 5) with increasing shear rate, which may be related to the characteristics of the membrane fouling layer: the turbulence caused by stirring may only break the accumulated particle layer with weak density or adhesion, and yet had little effect on the one with high adhesion on the membrane surface. Therefore, further increase in the shear rate could no longer effectively reduce the total resistance.

Figure 6b illustrates that the increase in TMP magnified the total resistance, which was most outstanding at the beginning of filtration (0–10 min). It can be attributed to the fact that the increase in transmembrane pressure may compress the contaminants on the membrane surface into a denser dirt layer, leading to the stronger filtration resistance [37]. Figure 6c shows that the steady-state $R_{f}$ of PVDF was generally higher than that of PES, especially when the shear rate was low (5.6 × 10^2^ s^−1^). Similarly, Figure 6d shows that the steady-state $R_{f}$ of PVDF was higher than that of PES no matter which TMP it was under. These results imply that the total process resistance was dependent on the membrane material, and PES showed better anti-resistance feature than PVDF in treating tanning wastewater, which could be attributed to the difference of membrane hydrophobicity: the organic foulants of tanning wastewater were more probable to foul a hydrophobic surface (PVDF material) rather than a hydrophilic one (PES material).

**Figure 7:**
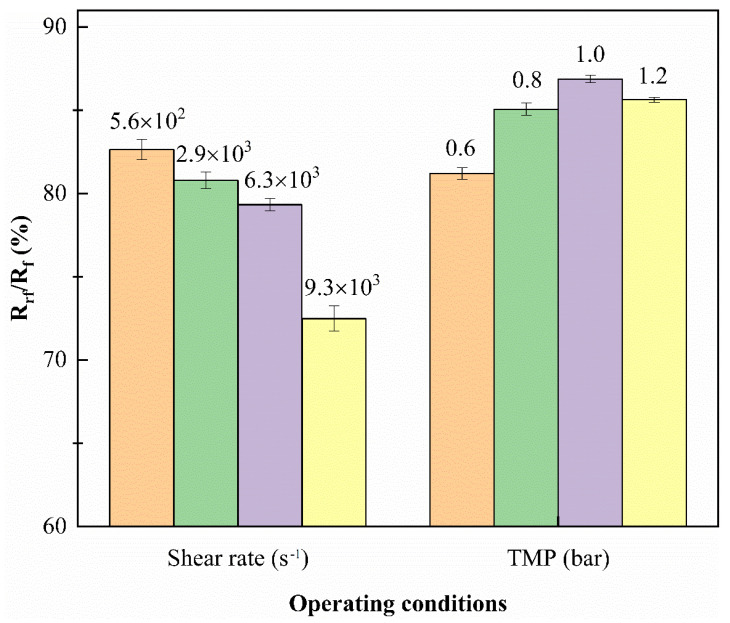
Percentage of the reversible fouling resistance ($R_{rf}$) in in total resistance ($R_{t}$).

The distribution of filtration resistance, under different operating conditions, is shown in Table 4. It can be observed that the reversible fouling resistance ($R_{rf}$) was the main source of resistance in the ultrafiltration process. Figure 7 shows that the increase in the shear rate reduced the proportion of reversible membrane fouling, indicating that the decrease in the total resistance, caused by the increase in the shear rate, was mainly due to the breakage of the reversible membrane fouling layer. In addition, the $R_{rf}/R_{t}$ also showed an increasing trend as the TMP increased, one possible reason is that higher TMP provided greater filtration driving force, and the filtered material liquid per unit time was increased, which increased the number of particles trapped on the membrane surface [38,39].

### 3.4. Fouling Propensity

**Figure 8:**
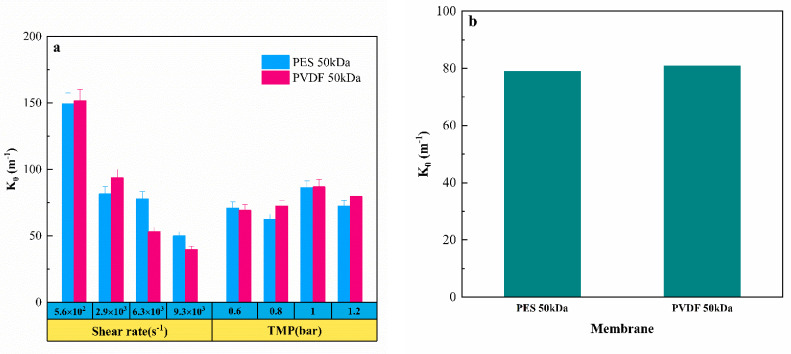
(**a**) Exponential fouling coefficient ($K_{0}$) of two membranes in different operating conditions; (**b**) the $K_{0}$ mean values of the two membranes under different operating conditions.

The fouling propensity coefficient ($K_{0}$) was calculated by Equation (12) via fitting the filtering experimental data accordingly. Figure 8a shows that the exponential fouling coefficient ($K_{0}$) of the two membranes under different operating conditions. The change of TMP had little effect on the growth rate of the membrane fouling layer, although the total filtration resistance continued to increase as the increase in the TMP, which means that the degree of contamination (density and thickness) of the pollution layer was constantly increasing, and TMP did not impact the accumulation rate of the pollution layer. In contrast, the increase in the shear rate reduced the accumulation rate of the fouling layer, and effectively enhanced the anti-fouling ability of the membrane, since $K_{0}$ decreased with the strengthening of shear rate. Figure 8b compares the $K_{0}$ mean values taking into account all the various conditions for the two different membranes, and it is found that there was not a significant difference between them, implying that the fouling growth kinetic did not depend on the membrane material in the process of tanning wastewater ultrafiltration.

### 3.5. Pore Blocking Mechanism

This section adopts the famous Hermans and Bredee model and its derivations (Equation (13) and Table 3) to interpret the pore blocking mechanism of the ultrafiltration process, providing further evidence for the fouling behavior.

#### 3.5.1. Effect of Membrane Material on Pore Blocking Mechanism

**Figure 9:**
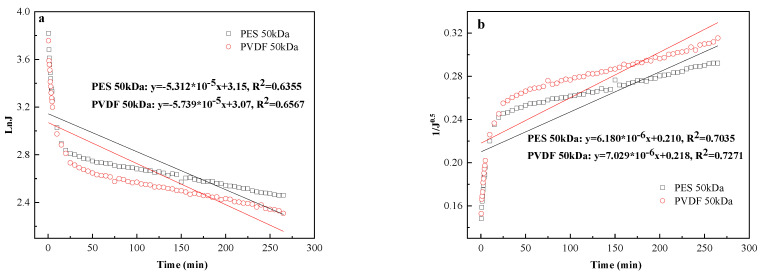

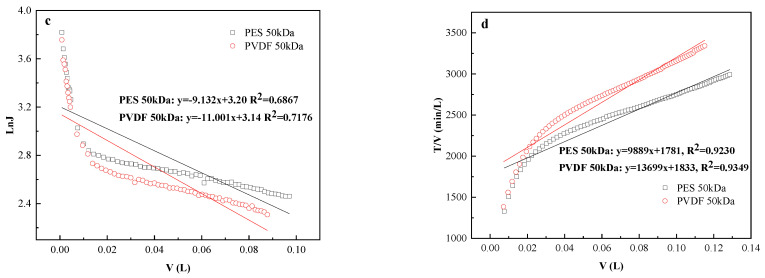
Pore blocking models fitting of tanning wastewater ultrafiltration: (**a**) complete blocking; (**b**) standard blocking; (**c**) intermediate blocking; (**d**) cake filtration (operating condition: 1.0 bar, 2.9 × 10^3^ s^−1^, 20 °C).

Figure 9a–d stands for complete blocking, standard blocking, intermediate blocking, and cake filtration, respectively. It can be found that the correlation coefficients of cake filtration fitting the curve were higher than 0.92 for both membranes, while the fitting degrees of the other three blocking models were low, and there is no obvious difference between the two membranes. Such results suggest that cake filtration was the main fouling mechanism in such ultrafiltration process.

It is worth noting that the initial data of cake filtration had a low degree of fit, which is speculated that the ultrafiltration process was not determined by a single membrane pore blocking behavior, but was more likely to be a synergy of multiple blocking behaviors. This phenomenon was consistent with another study [40].

#### 3.5.2. Effect of Operating Conditions on Pore Blocking Mechanism

**Figure 10:**
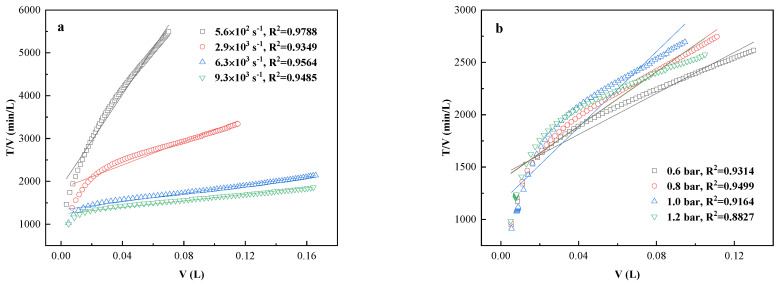
Data fitting of cake filtration model: (**a**) different shear rates (PVDF, TMP= 1.0 bar); (**b**) different TMP (PVDF, shear rate = 2.9 × 10^3^ s^−1^).

The section studies the influence of different operating conditions on the mechanism of membrane pore blocking. Figure 10 shows the result of utilizing the cake filtration model to fit the experimental data. The linear correlation coefficients ($R^{2}$) of such model were all above 0.88, which suggests that the operating conditions did not change the main fouling mechanism, and cake deposit was always the dominant foulant.

**Figure 11:**
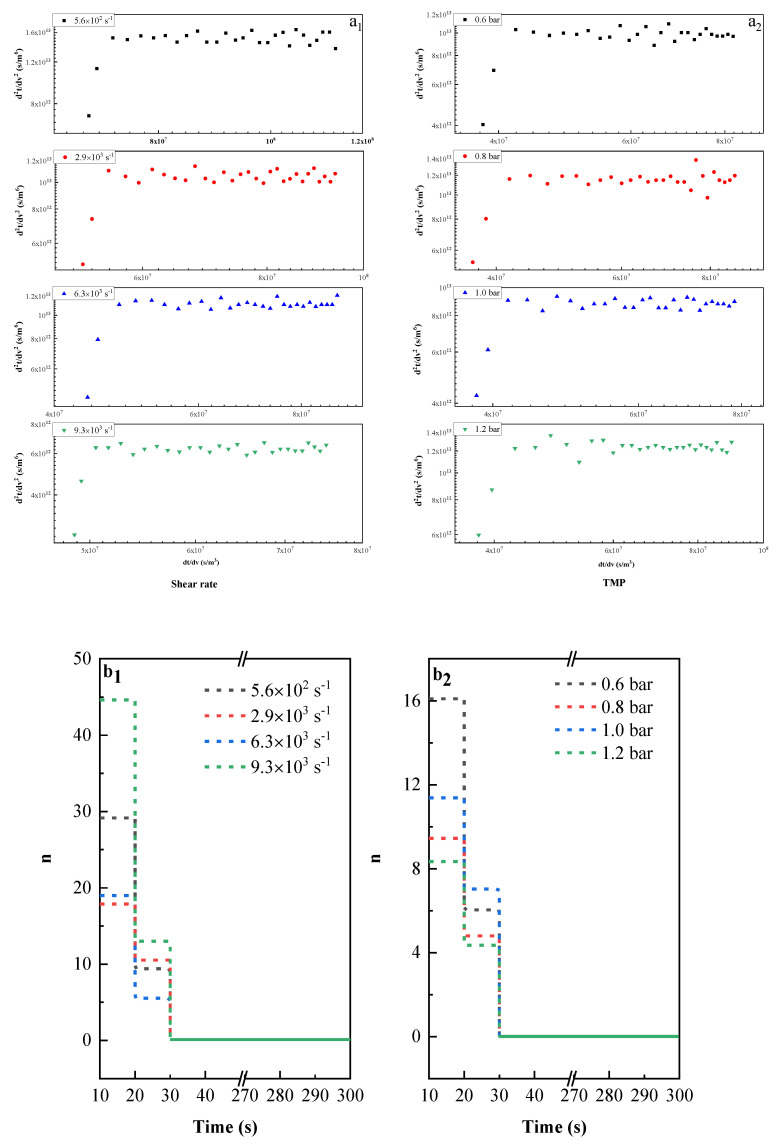
Fitting of experimental data at early stage of ultrafiltration (up to 5 min) with Herman′s model using equation $\frac{d^{2}t}{dv^{2}}=K{\left(\frac{dt}{dv}\right)}^{n}$ at different conditions (**a_1_**,**a_2_**); the evolution of blocking coefficient (n ) over time for different conditions (**b_1_**,**b_2_**). PVDF 50kDa was used in this section.

Figure 11 illustrates the evolution of the membrane pore blocking mechanisms over time at the initial stage of filtration (0–5 min). The experimental data at early stage was firstly fitted using Equation (13) (Figure 11(a_1_,a_2_)), the time interval of each data point in the figure was 10 s, the evolution of blocking coefficient n over time was then plotted in Figure 11(b_1_,b_2)_. It is found that the evolution of the blocking coefficient n, under different operating conditions, was almost the same: no matter which operating conditions, 30 s after the start of filtration, the slope of the curve decreased to 0 with time rapidly, which means that the ultrafiltration process reached the cake formation regime quickly.

## 4. Conclusions

In this work, the tanning wastewater was pretreated by ultrafiltration, high removal efficiency of SS, fat, and protein was revealed. The effect of membrane material, shear rate, and TMP on the pollutant removal efficiency, permeation efficiency, filtration resistance, fouling propensity, and pore blocking mechanism were assessed. Generally, the process efficiency strongly depended on the operating conditions, while the membranes of either PES or PVDF showed similar filtration performance and fouling behavior. Reversible resistance was the main obstacle for such a process. Cake formation was the main pore blocking mechanism during such a process, which was independent on the operating conditions and membrane materials. Moreover, there is nearly no transition of the pore blocking mechanism, the process reached the cake formation mechanism only after 30 s from its start.

The shear rate had a strong impact on the filtration efficiency and the removal efficiency: the increase in shear rate significantly increased the steady-state permeation flux, thus improved the filtration efficiency, which resulted from both the reduction in reversible resistance and the slow-down of fouling layer growth. However, the excessively high shear rate did not bring infinitive benefit for the anti-fouling purpose, which even led to a decrease in pollutant removal efficiency.

Compared to the shear rate, TMP had limited impact on the filtration behavior: the increase in TMP did not improve the filtration efficiency but increased the filtration resistance, especially the reversible one, while the growth rate of fouling layer stayed constant.

## Figures and Tables

**Table 1:** Characteristics of the tanning wastewater.

Parameter	Unit	Value
pH	-	5.11
Chemical oxygen demand (COD)	mg/L	21,305 ± 150.58
Fat	mg/L	1297 ± 38.30
Suspended solids (SS)	mg/L	4831 ± 131.16
Protein	mg/L	675 ± 5.93
Conductivity	mS/cm	38.67 ± 0.51

**Table 2:** Characteristics of UF membranes.

Designation	PES 50 kDa	PVDF 50 kDa
Manufacturer	SEPRO (USA)	Synder (USA)
Polymer type	Poly ether sulfone	Poly vinylidene fluoride
Molecular weight cut-off	50 kDa	50 kDa
Operating pressure	<6 bar	<8 bar
Operating pH	4–10	2–10
Maximum temperature	60 °C	60 °C

**Table 3:** Four ratiocinative models of Hermia blocking model.

Pore Blocking Models	Model Equations	Physical Concept
Complete blocking (*n* = 2)	$J=J_{ini}e^{-K_{b}t}$	Formation of a surface deposit
Standard blocking (*n* = 1.5)	$J=\frac{J_{ini}}{{\left(\frac{k_{s}J_{ini}}{2}t+1\right)}^{2}}$	Pore adsorption
Intermediate blocking (*n* = 1.5)	$J=J_{ini}e^{-K_{i}v}$	Pore constriction + surface deposit
Cake filtration (*n* = 0)	$\frac{t}{v}=\frac{K_{c}}{2}v+\frac{1}{J_{ini}}$	Pore blocking + surface deposit

**Table 4:** Decomposition of fouling resistance in different operating conditions (PVDF 50 kDa).

Operating Conditions		Fouling Resistance (×10^10^ m^−1^)			
		$R_{m}$	$R_{t}$	$R_{rf}$	$R_{irrf}$
Shear rate	5.6 × 10^2^ s^−1^	236	5650	4669	745
	2.9 × 10^3^ s^−1^	181	2269	1833	254
	6.3 × 10^3^ s^−1^	168	2057	1499	391
	9.3 × 10^3^ s^−1^	226	1841	1335	281
TMP	0.6 bar	109	1503	1220	174
	0.8 bar	158	2313	1968	188
	1.0 bar	162	2879	2501	216
	1.2 bar	186	2965	2539	240

## Data Availability

All data presented in this study are available in the current article.
